# Supplementary material for: CCL4 Regulates Eosinophil Activation in Eosinophilic Airway Inflammation
Source: Int J Mol Sci. 2022 Dec 18;23(24):16149. doi: 10.3390/ijms232416149 (PMC9782438; doi:10.3390/ijms232416149)
Supplement: Supplementary file 1 [file ijms-23-16149-s001.zip › ijms-2052607-supplementary.pdf]

Table S1. Amplification primers (5′–3′)

**gene**	**forward**	**reverse**
CCL4	CTC CTC ATG CTA GTA GCT GCC TTC	GGT GTA AGA AAA GCA GCA GGC GGT
CCL5	GTT GCA CCA AGC TAT GCA GGT	GCA GAA GCG TTT GGC AAT GT
CCL11	CCA CAC TGA AGG TCT CCG CA	TCT CTA GTC GCT GAA GGG GT
CCL26	ACT CCG AAA CAA TTG TGA CTC AGC TG	GTA ACT CTG GGA GGA AAC ACC CTC TCC
CCR5	CCC AGT GCA CAC AAG TGT AGG TAT C	GCT CAA GGC AGC TTA TTT CCA A
GAPDH	TTC ACC ACC ATG GAG AAG GC	AGG AGG CAT TGC TGA TGA TCT

Table S2. Patients' characteristics

	ECRS		Non-ECRS (n=5)
	rich Eos (n=5)	poor Eos (n=5)	
Age	56.0±6.9	50.8±15.4	62.4±11.7
Gender (M : F)	3 : 2	5 : 0	4 : 1
Eos in tissue (/HPF)	364 ± 172	32 ± 30**	8 ± 13**
Eos in blood (/μL)	529 ± 329	146 ± 44*	186 ± 214
FENO (ppb)	59.6 ± 18.4	20.4 ± 9.8**	NA
Polyp score	5.2 ± 1.6	4.4 ± 1.7	3.0 ± 2.0
LMS	15.4 ± 3.2	14.6 ± 6.9	10.4 ± 5.2
Total IgE (IU/mL)	159 ± 59	282 ± 474	982 ± 1864

ECRS; eosinophilic chronic rhinosinusitis, Eos; eosinophil, FENO; fractionated exhaled nitric oxide, HPF; high-power field, LMS; Lund-Mackay scale, NA; not applicable, Descriptive statistics are expressed as means ± SD, * $P < 0.05$, ** $P < 0.01$ (vs. ECRS with rich Eos).

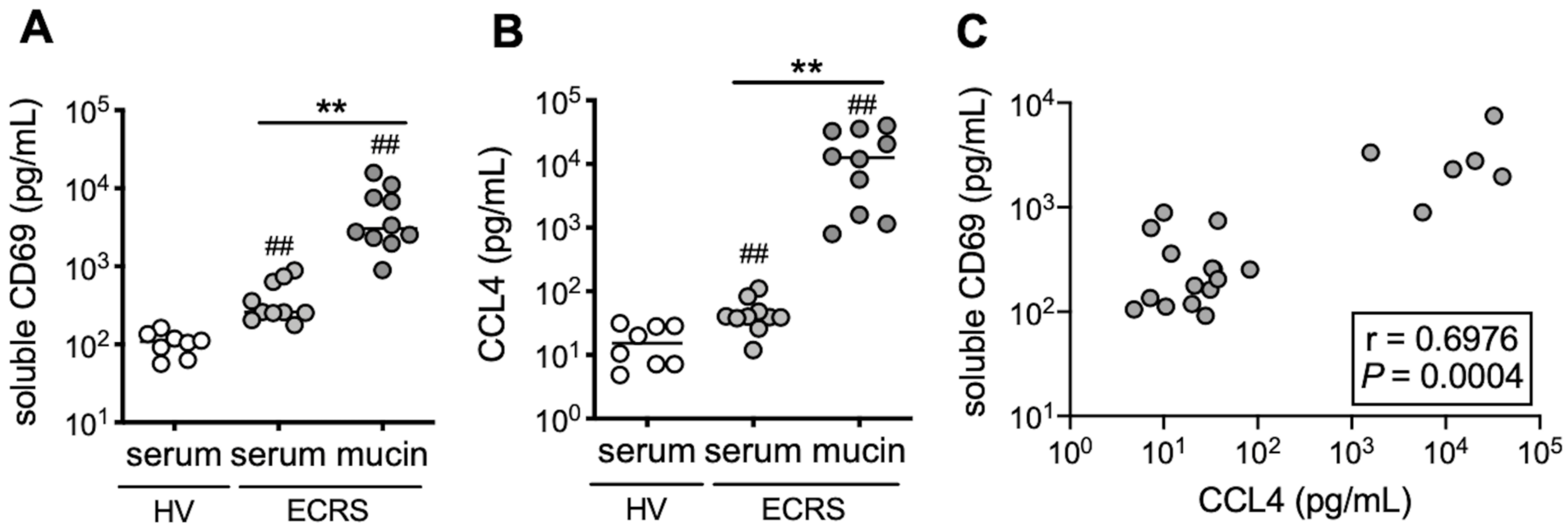

Figure S1. Soluble CD69 and CCL4 levels in serum and mucin of patients with refractory ECRS. **A, B,** Soluble CD69 (A) and CCL4 (B) levels in serum and/or mucin supernatant obtained from healthy volunteers (HV) and patients with refractory ECRS (ECRS) were measured. Individual values of serum from HV (n = 8), serum from ECRS (n = 10) and mucin from ECRS (n = 10) are shown; $^{\#\#}P < 0.01$ (vs. serum from HV), $^{**}P < 0.01$ (as shown between the two groups). **C,** Correlation between soluble CD69 and CCL4 levels (n = 21).

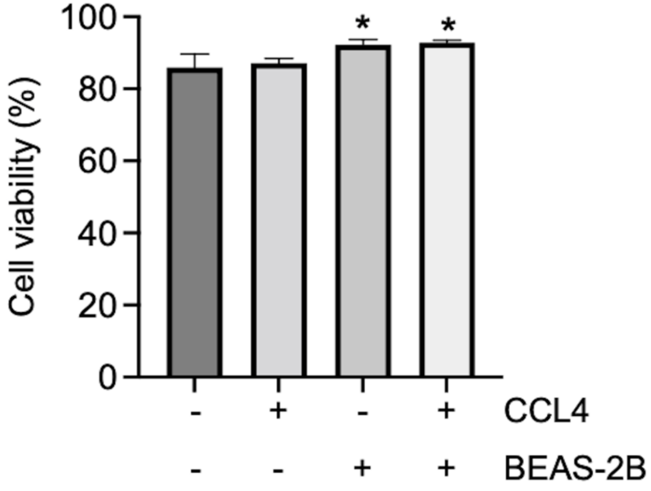

Figure S2. The effect of CCL4 on eosinophil survival. Viability of eosinophils coincubated overnight with BEAS-2B cells and/ or CCL4 (10 μg/mL) were analyzed by Flow cytometry. Values represent the mean ± SEM values of four experiments; **p* < 0.05 (vs. eosinophil without BEAS-2B co-incubation).
